# Chylous ascites after donor nephrectomy: MR lymphangiography and lymphatic embolization treatment

**DOI:** 10.1016/j.radcr.2022.12.013

**Published:** 2023-01-05

**Authors:** Tran Quoc Hoa, Nguyen Ngoc Cuong, Thieu Thi Tra My, Le Tuan Linh, Le Hoan, Pham Hong Canh, Trieu Quoc Tinh, Tran Nguyen Khanh Chi, Doan Tien Luu, Hoang Long

**Affiliations:** a Urology Surgery Department, Hanoi Medical University Hospital, Hanoi, Vietnam; b Diagnostic Imaging and Interventional Radiology Center, Hanoi Medical University Hospital, Hanoi, Vietnam; c Respiratory Department, Hanoi Medical University, Hanoi, Vietnam

**Keywords:** Chylous ascites, Living donor nephrectomy, Lymphatic embolization, Lymphangiography

## Abstract

Chylous ascites results from the leakage of lipid-rich lymphatic fluid into the peritoneal cavity. Most postsurgical chylous ascites occurs following abdominal aortic surgeries. However, rarely, it is a complication after laparoscopic donor nephrectomy. Postsurgical chylous ascites are often managed with conservative treatment or surgery, but lymphatic embolization may be required. Here, we presented a 45-year-old male patient who was referred for abdominal distension for 1 week after left donor nephrectomy. The drain fluid was milky and fluid analysis revealed high concentrations of triglycerides and chylomicron, confirming diagnosis of chylous ascites. The patient was treated with conservative therapy including a low-fat diet and fluid drainage but continued to have high draining output (up to 1500-2000 mL/24 h). He underwent magnetic resonance lymphangiography and intranodal lymphangiography, revealing extravasation of contrast into the abdomen and the left renal fossa. We embolized the interstitial lymphatic of the left retroperitoneal and lymphatic vessels leak. The patient was discharged from hospital at the fifth day after intervention. In this article, we demonstrate lymphatic lesions, the safety, and success of this technique.

## Introduction

Chylous ascites (CA) may result from traumatic or non-traumatic causes. The non-traumatic cause includes congenital defects of the lymphatic system, infections, malignancy, and increased peritoneal lymphatic pressure secondary to obstruction [1,2]. CA may follow abdominal aortic surgery and retroperitoneal lymph node dissection, and rarely after donor nephrectomy which damages the cisterna chyli or lymphatics tributaries [1]. The diagnosis of CA is based on the presence of a triglyceride level higher than 110 mg/dL [3]. There is no standard treatment for chylous ascites. Most cases of traumatic CA are managed with conservative treatment including dietary modification, total parenteral nutrition [4]. Interventional radiology and surgical treatment are generally recommended if conservative management is unsuccessful. We present a case of uncontrollable CA that failed with conservative therapy and was successfully treated by lymphatic embolization. This report highlights a rare complication of left donor nephrectomy and describes the lymphatic embolization approach based on identifying the exact leaking lymphatic channels.

## Case report

A 45-year-old male patient with no medical history suffered abdomen distension 1 week after left open donor nephrectomy for his wife. He found massive ascites which the paracentesis fluid sample was confirmed as chylous fluid because of its milky color and high concentration of triglyceride and chylomicron. The diagnosis was postoperative chylous ascites and the initial treatment was ascite drainage with total parenteral nutrition. For 10 days, the amount of drainage fluid remained from 1.5 to 2 L/d. The patient was referred to our hospital.

**Fig. 1:**
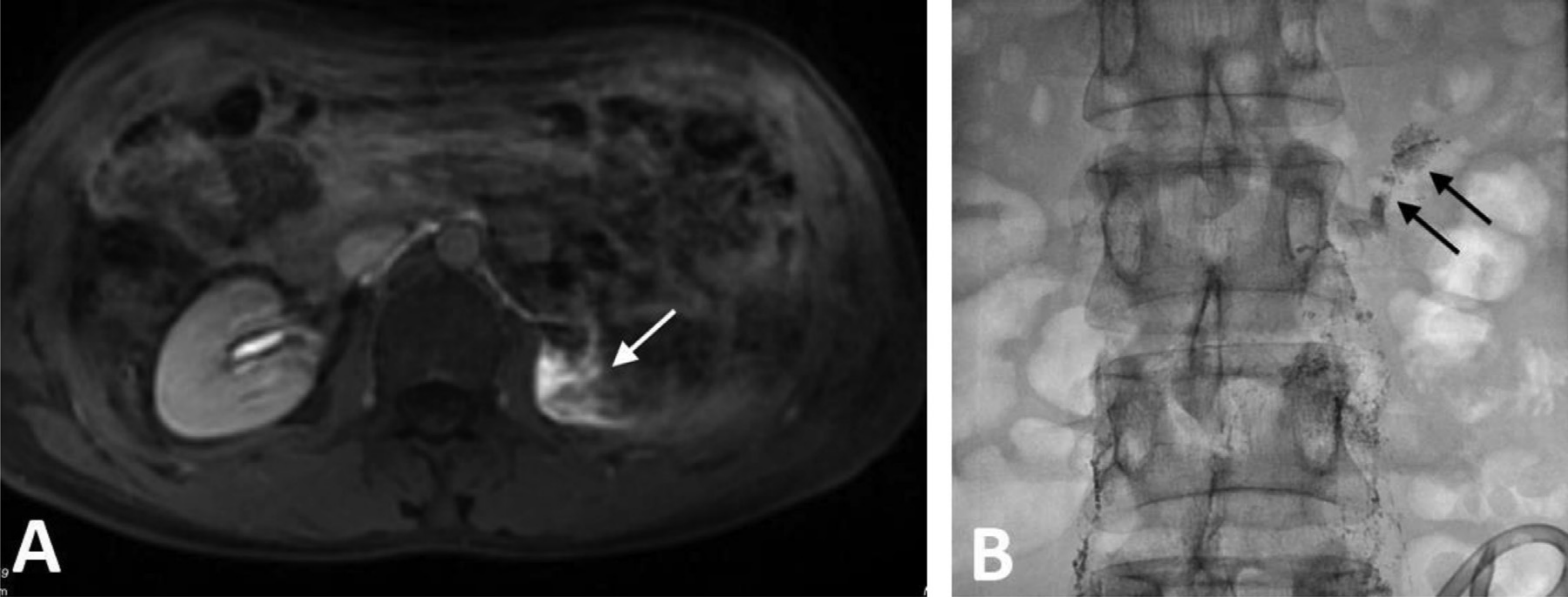
Extravasation of contrast was seen on MR lymphangioraphy and DSA. (A) The contrast was present at the left renal fossa (arrow). (B) DSA image: the extravasation in the left renal fossa presented before the appearance of cisterna chyli.

**Fig. 2:**
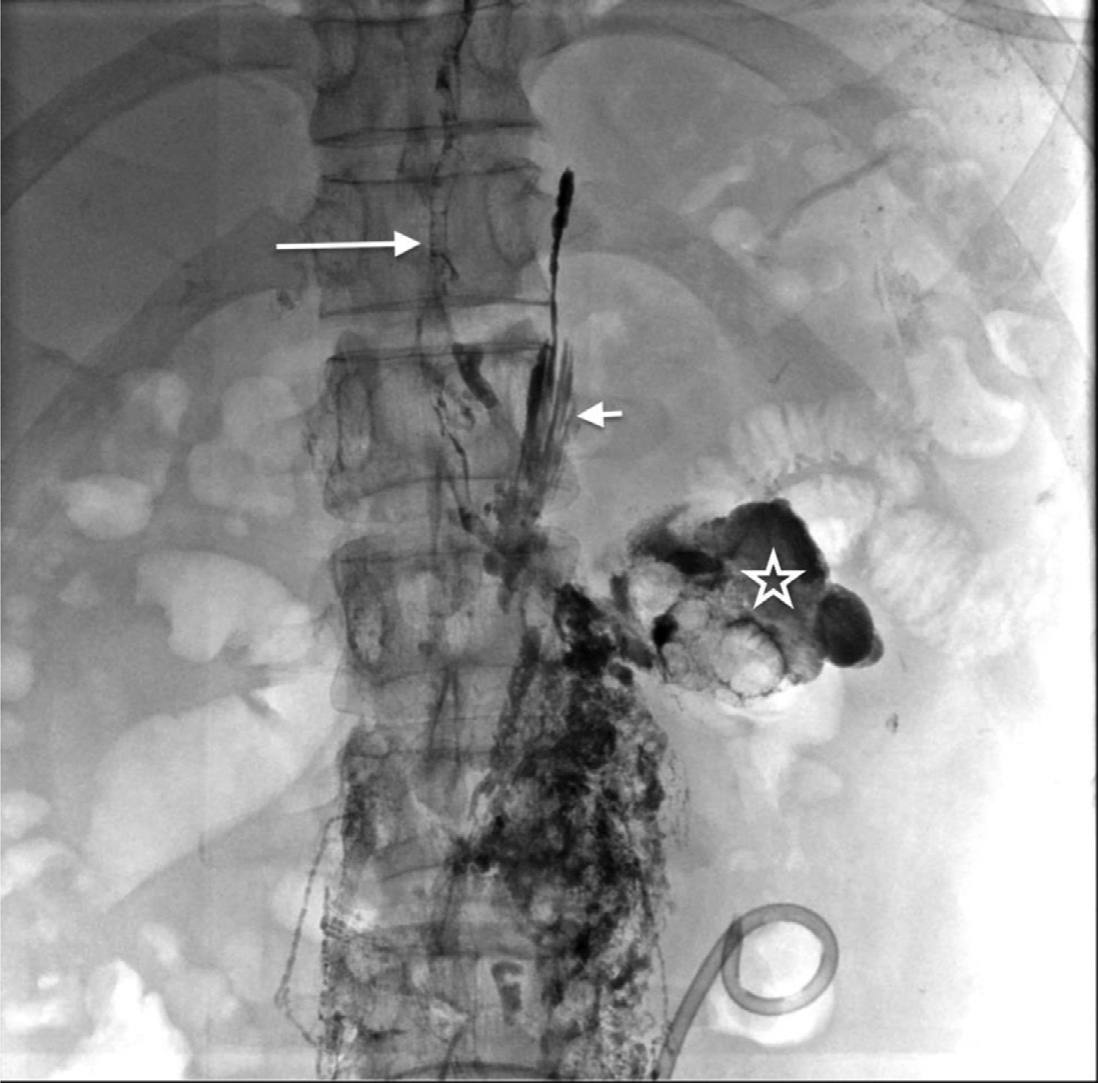
DSA image after embolization. The glue was in the lower part of thoracic duct (long arrow) and in the left renal fossa (star) which mean the fistular was occluded. The glue was leak from the thoracic duct at the end of injection (short arrow) without consequence.

**Fig. 3:**
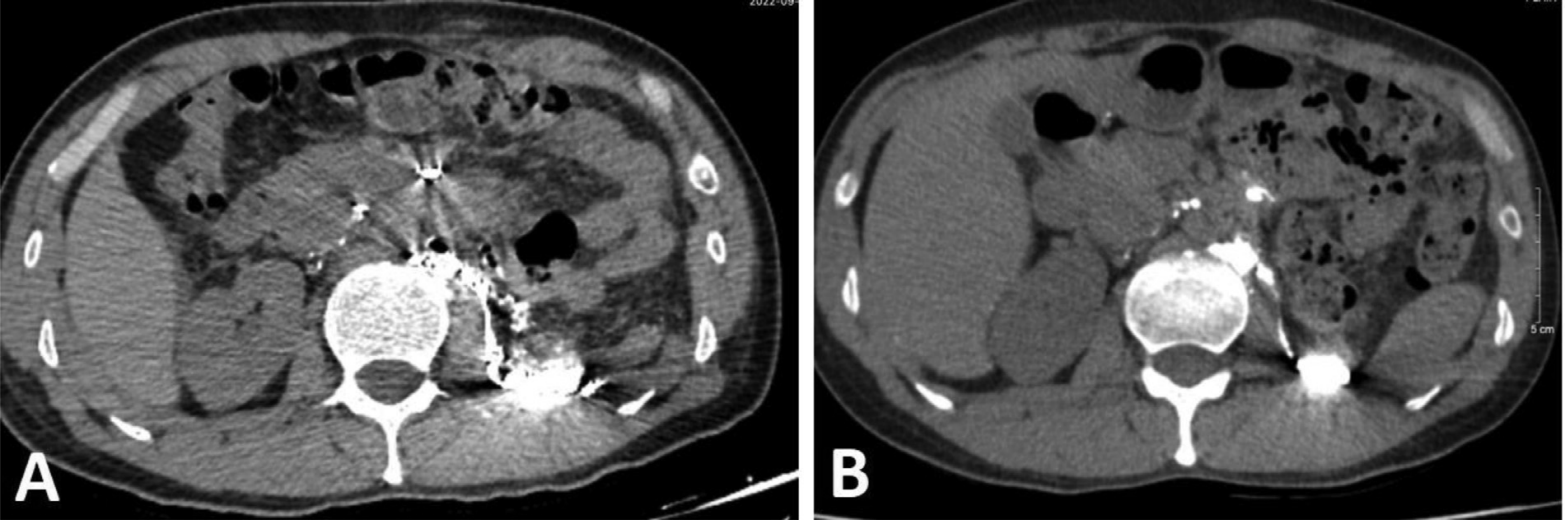
Abdomen CT scan after intervention (A) and 3 months after discharge (B) showed deposition the glue cast of the lymphatic vessel and the left renal fossa.

Because the diagnosis was postoperative CA, we performed magnetic resonance (MR) lymphangiography which is the routine imaging modality for patients with postoperative chylous leaks in our institution. On MR lymphangiography, there was extravasation of contrast agent into the abdomen and the left renal fossa (Fig. 1A). The thoracic duct was not seen on the MR lymphangiography. The patient was then indicated to do intranodal lymphangiography. On lymphangiography, there was an extravasation of the contrast agent into the left renal fossa in early phase before the appearance of cisterna chyli (Fig. 1B). When the cisterna chyli was visible, we found that there was stagnation of contrast agent without flowing into the thoracic duct. We assumed that there might be an occlusion of thoracic duct causing the reflux of chyle into the renal fossa. We punctured the cisterna chyli (Chiba, 21G) and injected 2.5 mL glue to block the backflow of the lymphatic vessels. During injection, we noticed the glue went into the thoracic duct and refluxed into the left renal fossa. Additionally, the interstitial lymphatic vessels at lumbar region were also punctured (Chiba 21G) to be embolized by glue NBCA (2 mL mixture of NBCA: lipiodol, 1:4), blocking the afferent lymphatic vessel into the left renal fossa (Fig. 2). The non-contrast computed tomography (CT) scanner after intervention confirmed the cast of glue in the left renal fossa (Fig. 3A).

After intervention, the drain out with 700 mL of reddish fluid which there contained blood. The day after the fluid decreased significantly to about 10 mL. The patient came back to a normal diet on the third day after intervention. On the fifth day after intervention, the drain was withdrawn and the patient was discharged from hospital. Follow-up abdominal CT scanner after 3 months showed no ascites; the glue cast of the lymphatic vessel and the left renal fossa remained unchanged (Fig. 3B).

## Discussion

Postsurgery chylous ascites is a rare, but potentially life-threatening complication following abdominal surgery. Up to 80% of all postsurgical CA results from aortic repair, followed by retroperitoneal lymph node dissection, liver transplant, and donor nephrectomy [4]. A review of Veracierto et al. documented 62 cases of CA after donor nephrectomy [5]. The incidence of CA after donor nephrectomy ranges between 0% and 1.83% [6]. This complication is more common after left donor nephrectomy than right [6,7]. The exact cause remains unclear; however, it could be explained that the frequency of left donor nephrectomy is much greater than the right. The other reason that can explain the different incidences of 2 sides was the anatomic factors of the left renal artery and lymphatic system [7]. The lumbar lymphatic trunks and the cisterna chyle are closely related to the aortic artery. Moreover, the left renal artery is usually considerably shorter than the right; therefore, ligation of the left renal artery close to the aorta has a high risk of damaging to the cisterna chyli and its tributaries.

Patients commonly become symptomatic as soon as they resume oral intake, ranging from weeks to months because of rupture of the lymph vessels or adhesions of the lymph vessels [5,8]. Patients may present with abdominal distention, weight gain, and dyspnea. Abdominal paracentesis helps confirming chylous ascites. The fluid will be milky with high triglyceride content. Lymphangiography not only could provide the definitive diagnosis of chylous ascites but also can pinpoint the exact site of chyle leak [4]. In our patient, on both MR lymphangiography and digital subtraction angiography images, the thoracic duct did not appear until we punctured the cisterna chyli and embolization the leakage. The stagnation in the thoracic duct may cause collateral circulation of lymphatic vessels at the lumbar region and may lead to abnormal lymphatic circulation around the left renal hilar. Chronic thoracic duct obstruction may cause abnormal lymphatic circulation at the renal pelvis, so any cut made by operation at this region may damage the lymphatic vessels. This phenomenon is also observed in some patients with chyluria in which the thoracic duct is obstructed, and abnormal dilated lymphatic vessels present at the renal hilar [9,10].

Some cases with CA may spontaneously resolve without any treatment because a small leakage site of the lymphatic channel can close spontaneously. Approximately 77% of cases could be successfully managed conservatively [7]. However, patients who had high-output CA need to be hospitalized for treatment. Surgical repair may be indicated for patients who do not respond to conservative treatment. Recently, some radiological intervention techniques are used to control lymphatic leakage. Itou et al. [11] and Hargis et al. [12] treated successfully CA with percutaneous embolization of the leaking lymphatics from outside the lymph vessel under CT guidance. In the study of Hiffa et al. [1], the patient was successfully treated with intranodal lymphangiography embolization of the left retroperitoneal chylous leak under ultrasound guidance. In this patient, we embolized the reflux of chyle from the cisterna chyli and then the afferent lymphatic vessels at the lumbar to ensure the permanent occlusion of chylous fistular. Application of non-invasive imaging modality and lymphatic intervention in postoperative CA is well known recently and its efficacy is proven. There is clear correspondence in detecting the ruptured vessel between MR image (Fig. 1A) and the glue cast in the CT scanner after embolization (Fig. 3).

## Conclusion

Chylous ascites is a rare complication of donor nephrectomy. Thoracic duct occlusion and hyper lymphatic vascularity collateral at the lumbar may increase the risk of such complication. Lymphatic embolization may be feasible for the treatment of massive or refractory chyle leakage after nephrectomy.

## Author contributions

The authors confirm contribution to the paper as follows: study conception and design (Nguyen Ngoc Cuong, Tran Quoc Hoa), data collection (Nguyen Ngoc Cuong, Pham Hong Canh), draft manuscript preparation (Thieu Thi Tra My, Nguyen Ngoc Cuong, Tran Nguyen Khanh Chi, Le Hoan, Le Tuan Linh, Doan Tien Luu, Hoang Long). All authors reviewed the results and approved the final version of the manuscript.

## Patient consent

Patient was informed that his documents (without personal information) including diagnosis and treatment information might be published for science purpose. He agreed and signed in the consent form.
